# Analysis of the Reasons for Transferring Patients from Orthopedic Specialty Hospitals to Tertiary Hospitals After Surgery

**DOI:** 10.3390/jcm14144943

**Published:** 2025-07-12

**Authors:** Juneyoung Heo, Su Chan Lee, Ji Hyun Kim, Chang Hyun Nam, Dong Nyoung Lee, Ji-Hoon Baek

**Affiliations:** 1 Joint & Arthritis Research, Department of Neurosurgery, Himchan Hospital, Seoul 07999, Republic of Korea; 2 Joint & Arthritis Research, Department of Orthopaedic Surgery, Himchan Hospital, Seoul 07999, Republic of Korea

**Keywords:** orthopedic, specialty hospital, transfer, pulmonary embolism, cholecystitis

## Abstract

**Background**: This study investigated the reasons for transferring patients to tertiary hospitals due to complications arising after surgery at orthopedic specialty hospitals and the treatment results. **Methods**: This retrospective cohort study was conducted on all orthopedic patients, except for the spine, at a single institution from January 2012 to May 2022. **Results**: Of 67,118 patients, 167 (0.24%) were transferred to a tertiary hospital after surgery. Patients’ average age was 72.2 ± 9.2 years (range: 28–91 years), and there were 34 men and 133 women. The most common reason for transfer to a tertiary hospital was pulmonary embolism (27 patients, 16.2%), which occurred on an average 3.81 days (range: 0–23 days) after surgery, and 25 patients were transferred within 1 week after surgery. The next most common cause was acute cholecystitis (19 patients (11.4%), all of whom had undergone total knee arthroplasty), which occurred on an average 6.15 days (range: 1–14 days) after surgery. **Conclusions**: The rate of transfer from the orthopedic hospital where this study was conducted to a tertiary hospital was very low at 0.24%. Pulmonary embolism, acute cholecystitis, and cerebral infarct were the most common reasons for transfer. In particular, acute cholecystitis was the second most common reason for transfer, and caution should be taken because elderly patients may experience only atypical clinical symptoms without abdominal pain or tenderness even the day after surgery.

## 1. Introduction

Specialized hospitals originated in the United States in the 1800s [1], after which their number has been gradually increasing [2]. Unlike general hospitals, specialized hospitals focus on treating specific medical conditions, saving time and simplifying procedures, thereby enabling efficient treatment [2]. To promote specialized and efficient treatment as an alternative to the increase in the incidence of chronic diseases and the rising medical expenses due to the aging population, South Korea has designated and operated specialized hospitals since 2011, with the largest number of hospitals being orthopedic specialty hospitals.

Orthopedic specialty hospitals have several advantages, such as shorter hospital stays, lower readmission rates, and better clinical outcomes [3,4,5], and this is true not only for specialized hospitals specializing in joint replacement surgery [6,7] but also for spine specialty hospitals [8]. Nevertheless, as the elderly population increases, the risks before and after surgery increase, which can be a burden for specialized hospitals. In particular, knee and hip joint replacement surgeries are often performed in the elderly population, and as they are more invasive and cause more bleeding than other surgeries, the incidence of postoperative complications in elderly patients may be higher than that in other surgeries. When complications occur in areas other than their specialty, specialized hospitals transfer patients to tertiary hospitals for treatment because of the difficulty of treating them. If the occurrence of postoperative complications can be analyzed and predicted, it would be possible to offset the shortcomings of specialized hospitals by responding more quickly and efficiently when complications occur.

Therefore, we investigated the reasons for transferring patients to tertiary hospitals due to complications arising after surgery at orthopedic specialty hospitals and the treatment results. We anticipate that this study will be helpful for safely and efficiently managing complications arising at orthopedic specialty hospitals.

## 2. Materials and Methods

This retrospective cohort study was conducted on all orthopedic patients, except for the spine, at a single institution from January 2012 to May 2022. The design and protocol of this retrospective cohort study had been approved by the Institutional Review Board of our hospital, who waived the requirement for informed consent. Our orthopedic specialty hospital has the capacity to perform procedures for most orthopedic disorders, including joint replacement. Consultations are available from internal medicine, radiology, neurology, pain medicine, and anesthesiology specialists for preoperative evaluation and postoperative management; however, intensive treatment is not possible with subspecialists, as in tertiary hospitals. Specialized nursing, physical therapy, and rehabilitation treatment are available, and a doctor on duty is always on standby in the hospital to respond to emergencies. In the following cases, the patients were judged as high-risk and were not treated at our hospital: (1) ASA PS (American Society of Anesthesiologists Physical Status) IV or more, (2) positive pregnancy test, (3) body mass index (BMI) > 40 kg/m^2^, (4) automatic implantable cardioverter defibrillator, and (5) personal or family history of malignant hyperthermia.

According to the recommendations of the American Academy of Orthopedic Surgeons guidelines (2011) [9] and the Korean guideline for the prevention of venous thromboembolism (2012) [10], rivaroxaban and mechanical compression devices were applied to all patients without contraindications for the prophylaxis of venous thromboembolism. Moreover, all patients undergoing lower extremity surgery underwent preoperative Doppler ultrasound examination of both lower extremities, and if a thrombus was detected in the deep vein, it was treated before surgery.

When postoperative abnormal symptoms occurred, all judgments were made in consultation with an internal medicine or neurology doctor to decide whether to transfer to a tertiary hospital. In total, 167 patients were transferred to a tertiary hospital during this cohort study period. During the COVID-19 pandemic period in the middle of this study period, treatment was conducted in strict compliance with the government’s quarantine guidelines. Only patients who underwent COVID-19 vaccination as recommended by the government underwent orthopedic surgery at our hospital, and patients infected with COVID-19 before surgery underwent surgery after being judged to have recovered. There were 11 cases of isolation or transfer due to COVID-19 infection during the postoperative hospitalization; however, because all patients recovered without any particular complications, they were not included in this study.

All patients were transferred to a tertiary hospital within 1 h from our hospital if postoperative complications developed and transfer was deemed necessary. If the situation was considered urgent, they were transferred to a tertiary hospital within 15 min. In particular, patients with conditions such as pulmonary embolism, ischemic heart disease, and acute cholecystitis that required intensive care unit treatment or could result in serious consequences if suddenly worsened were transferred to a tertiary hospital as soon as suspected. A medical treatment cooperation system has been established with hospitals to where patients can be transferred; hence, before transferring, we check whether the tertiary hospital has the capacity to provide treatment and then notify the patient’s information in advance before transferring, so that treatment is possible immediately upon arrival at the tertiary hospital. After the completion of the diagnosis and treatment at the tertiary hospital, the results were notified to our hospital.

We examined the demographic characteristics and comorbidities of patients who developed complications, including gender, age, and BMI. The cause of the transfer and the subsequent course of treatment were investigated based on the treatment results sent from tertiary hospitals and the charts treated at our hospital. In cases where patients died, autopsies were not performed, and the cause was estimated based on symptoms and results of tests performed before death.

Variables were analyzed using multinominal logistic regression and multivariate analysis of variance. All *p*-values were two-sided, with a *p* value of <0.05 indicating statistical significance. All analyses were performed using the Statistical Package for the Social Sciences version 27 (SPSS v27, Chicago, IL, USA).

## 3. Results

From January 2012 to May 2022, 67,118 orthopedic surgeries, excluding the spine, were performed in our hospital, which included 28,556 total knee arthroplasties (TKAs) and 42 total hip arthroplasties (THA). Other than these, shoulder surgeries were the most common, followed by ankle and foot and trauma surgeries (Table 1). Of the 67,118 patients, 167 (0.24%) were transferred to a tertiary hospital after surgery, including 34 men and 133 women with an average age of 72.2 ± 9.2 years (range: 28–91 years) and BMI values of 24.5 ± 3.5 and 25.1 ± 4.2 kg/m^2^ for men and women, respectively. The average Elixhauser Comorbidity Index of patients who underwent elective treatment was 1.64 ± 1.14 (Table 2). Most patients were transferred to tertiary hospitals between 2016 and 2019, after which the number gradually decreased (Table 3).

Among patients who were transferred, TKA was the most common, with 136 patients (81.4%), followed by THA with 11 patients (6.5%) and shoulder surgeries with 9 patients (Table 1). A total of 17 patients (10.2%) were transferred on the same day of surgery, with the most common causes being pulmonary embolism in five patients and cardiac complications in four patients (cardiac arrest in two patients and myocardial infarction in two patients). Other causes included vascular damage at the surgical site, cerebral infarction, gastrointestinal bleeding, and asthma attacks. Four patients were transferred 3 weeks after surgery, with the causes being surgical site infection in two patients, deep vein thrombosis in one patient, and pulmonary embolism in one patient.

Pulmonary embolism was the most common reason for transfer to a tertiary hospital (27 patients, 16.2%) (Table 4), which occurred on an average 3.81 days (range: 0–23 days) after surgery, and 25 patients were transferred within 1 week after surgery. Moreover, three patients died due to pulmonary embolism, and four patients developed severe sequelae due to pulmonary embolism and were lost to follow-up at our hospital. The remaining patients were treated well without any sequelae. Of the 27 patients with pulmonary embolism, 26 underwent TKA, and 1 underwent THR.

The next most common cause was acute cholecystitis, accounting for 19 patients (11.4%), all of whom had undergone TKA. All patients, except three, had right upper quadrant abdominal pain and tenderness. The three patients had no abdominal pain or tenderness, and all were elderly patients aged >70 years. One patient had only repeated vomiting without abdominal pain, another one had fever and an increase in C-reactive protein level, and the remaining one had only jaundice. All patients had acute cholecystitis that was confirmed by blood test results and abdominal ultrasound or computed tomography). Acute cholecystitis occurred an average 6.15 days (range: 1–14 days) after surgery, and all patients improved without any complications after cholecystectomy at a tertiary hospital.

The third most common cause was cerebral infarction, accounting for 17 patients (10.1%), and these patients were transferred to the hospital after an average 2.46 days (range: 0–7 days) post surgery. Of the 17 patients, 15, excluding 2, were transferred within 3 days after surgery. Although no patient died due to cerebral infarction, five patients suffered from sequelae, such as hemiplegia, paraplegia, and severe decline in consciousness and cognitive function, and were lost to follow-up at our hospital. Other common causes were respiratory distress (*n* = 9), myocardial infarction (*n* = 8), surgical wound problems (infection or redislocation) (*n* = 6), and myocardial infarction (*n* = 5).

When classified by system, the most common cause for transfer to a tertiary hospital was gastrointestinal disease, accounting for 35 patients (20.9%) (Table 5). There were 19 patients with acute cholecystitis, 4 with intestinal bleeding, 3 with intestinal obstruction, and 3 with appendicitis. Other causes included intestinal adhesions, peritonitis, and severe abdominal pain. The next most common cause by system was pulmonary thromboembolism, accounting for 34 patients (20.4%). Pulmonary embolism accounted for 27 patients, fat embolism accounted for 5 patients, and deep vein thrombosis accounted for 2 patients. The third most common cause by system was brain-related disease, which led to the transfer of 29 patients (17.4%). Cerebral infarction was the most common cause, accounting for 17 patients, followed by cerebral hemorrhage accounting for 7 patients, decreased consciousness accounting for 4 patients, and transient ischemic attack accounting for 2 patients. The fourth most common cause was cardiac disease, accounting for 24 patients (14.3%), which included myocardial infarction in 8 patients, chest pain in 4 patients, arrhythmia in 3 patients, and heart failure in 3 patients.

Despite transfer, 11 patients died, of whom 5 died due to sudden dyspnea and cardiac arrest. This phenomenon occurred on an average 3.4 days (range: 1–6 days) after surgery, and the patients died before the cause was identified. Moreover, because an autopsy was not performed after death, the exact cause remains unknown. Nevertheless, considering that cardiac arrest occurred within 10 min of the onset of symptoms, death occurred within 1–2 h, and the opinions of the internists who treated the patients, it is suspected that the death was due to heart problems or pulmonary embolism. The next most common cause of death was pulmonary embolism in three patients, myocardial infarction in two patients, and peritonitis in one patient. Of the 11 patients who died, 6 underwent TKA, 2 underwent shoulder arthroscopy, and 1 underwent THR. The average age of the patients who died was 69.3 years old (range: 28–87 years), and except for a 28-year-old woman, all the remaining patients were aged >58 years. The 28-year-old woman had undergone anterior cruciate ligament reconstruction surgery without any underlying disease, and 5 days after the surgery, she suddenly experienced cardiac arrest of unknown cause and was transferred to a tertiary hospital, but later died.

The most common days of the week for transfer to tertiary hospitals were Wednesday and Tuesday, with 36 (21.5%) and 30 (17.9%) patients, respectively. The lowest numbers were on Friday and Sunday, with 20 (11.9%) and 13 (7.7%) patients, respectively (Table 6). However, the highest number of deaths occurred on Friday and Sunday, with three patients each (27%).

Multivariate regression analysis was performed to identify independent predictors of patient transfer to a tertiary hospital. Accordingly, the presence of obesity was found to be an independent predictor of transfer to a tertiary hospital due to pulmonary thromboembolism and gastrointestinal system disease (*p* = 0.003, 0.002). Moreover, sex, age, BMI, and all comorbidities in the Elixhauser Comorbidity Index were not independent predictors of transfer.

## 4. Discussion

The most remarkable finding of this study is that the second most common reason for transfer to a tertiary hospital was acute cholecystitis that occurred in 19 patients (11.4%), all after TKA. The incidence of acute cholecystitis among patients who underwent TKA was 0.066%. Although there have been several case series reports on the occurrence of acute cholecystitis after orthopedic surgery before 2000, there were no specific statistical data, and it was known to occur rarely [11]. Incidence rates of cholecystitis after THA or hip surgery ranged from 0.1–0.7%, which can be considered relatively common compared to other orthopedic surgeries. A study by Ottinger LW et al. in 1976 found that 30% of patients who developed acute cholecystitis as a postoperative complication developed cholecystitis after THA [12], whereas in a nationwide cohort study by Jang SY et al. in 2019 revealed that the incidence of cholecystitis after hip fracture in elderly patients was 0.24% [13]. Studies by Choo SK et al. and Yuan Y et al. found that the incidence of cholecystitis after hip fracture was 0.74% and 0.13%, respectively [11,14]. Moreover, a study by Deleanu B et al. showed that cholecystitis developed in 2 out of 9268 patients who underwent hip surgery [15]. Overall, the occurrence of cholecystitis after TKA appears to be exceptionally rare, with only case reports by Wilde AH et al. in 1988 and Ghalimah B et al. in 2016 being available [16,17]. This phenomenon does not occur only in orthopedics but can also occur in various fields, including thoracic surgery and urology [18]. Previous studies reported that it occurred 3 days after surgery [15,18]; however, in the present study, it occurred on the day after surgery and 2 days after surgery. Moreover, considering that elderly patients often exhibit atypical symptoms, additional caution is required. In the present study, three elderly patients exhibited only nonspecific symptoms, such as vomiting, fever, and jaundice, without abdominal pain and tenderness, which are common symptoms of acute cholecystitis. In a previous study, 27% of patients with acute cholecystitis aged >70 years did not have right upper quadrant abdominal pain, and 45% did not have fever [19]. Given the results of these studies, it is necessary to consider the possibility of acute cholecystitis in elderly patients who develop atypical clinical behavior even on the day after surgery.

In this study, the most common reason for transfer from an orthopedic hospital to a tertiary hospital was pulmonary embolism, accounting for 27 patients (16.2%), which was a finding different from the results of other studies, wherein the most common reasons for transfer were respiratory disease in the study by Dawson et al. [20] and cardiac complications in the study by T. D’Amore et al. [21]. Pulmonary embolism has long been recognized as a major complication after lower extremity joint replacement surgery [22,23], and the fact that TKA and THA accounted for 42.6% of the surgeries performed in this study, which was higher than that in other studies, may have influenced the high incidence of pulmonary embolism. Furthermore, pulmonary embolism was likely to occur as a serious outcome, with three deaths and four fatal sequelae, all of which occurred within 1 week after surgery. The proportion of patients who underwent TKA or THA and were transferred to a tertiary hospital due to pulmonary embolism was 0.09%, which was significantly lower than that (0.2–0.5%) reported in previous studies [24,25]. The death rate due to pulmonary embolism among patients who underwent TKA in the present study was 0.028%, which was significantly lower than the rate of 0.37% reported in a 2012 meta-analysis [26]. A reason for the significantly lower incidence and mortality rate of pulmonary embolism in our study hospital may be the preoperative use of Doppler ultrasound for both lower extremities. A recent study by Li H et al. (2025) reported that performing perioperative ultrasound screening of lower extremity veins before orthopedic surgery reduced the incidence of pulmonary embolism and fatal pulmonary embolism [27]. Another reason may be that all patients visiting our hospital are Korean who have been reported to have a significantly lower rate of venous thromboembolism than other races [28,29]. One study found that the incidence of PE after TKA was lower in Asians than in other races [30]. In fact, a meta-analysis showed that the incidence of symptomatic PE in Asians was 0.01%, which was lower than that reported in studies involving other races, and that no difference existed among Asian countries [31].

The most common cause of death was probably pulmonary embolism or cardiac problems, which accounted for 10 of 11 patients in this study. No patient died due to cerebral infarction; however, five patients developed serious sequelae. These findings suggest that the most serious complications were pulmonary embolism, heart problems, and cerebral infarction, which were similar to those reported in other studies of orthopedic surgery. For instance, Memtsoudis et al. investigated perioperative mortality after lower extremity arthroplasty and reported that the complications with the highest mortality rate were pulmonary embolism and central nervous system and heart problems [32]. Belmont et al. also reported that cardiac complications were the most common cause of death after TKA, accounting for 44.6% [33].

The transfer rate of patients who underwent surgery to a tertiary hospital was very low at 0.24% in the present study. The transfer rate of patients who underwent joint replacement surgery was 0.51%, which was higher than that of patients who underwent other surgeries; this result was similar to the transfer rate of 0.62% after TKA and THA reported in the study by D’amore et al. [21]. In the present study, of all patients who underwent surgery, 42.6% underwent TKA and THA, whereas 88% were transferred to tertiary hospitals. Similarly, in a study by Dawson et al. (2015), 37.7% of all patients underwent TKR and THA, whereas 63.7% were transferred [20]. Thus, the results of the present study support those of previous studies that patients who underwent joint replacement surgery are more likely to be transferred to tertiary hospitals than those who underwent other orthopedic surgeries.

Patient transfers occurred most frequently on Wednesdays in the present study; however, there was no significant difference according to the day of the week. In contrast, other studies reported patient transfers primarily on weekends [20,21,34], which was different from the result of the present study. This difference may be because our study hospital arranged regular surgeries considering manpower arrangement according to time.

Changes in disease epidemiology occur only under specific circumstances and not under general conditions. For instance, during the COVID-19 pandemic, significant changes in the epidemiology of trauma were noted. Lockdown measures and difficulties in accessing health care have altered overall hospitalization and mortality rates, frequency of injuries to specific body parts, and mechanisms of injury [35]. Differences between rural and urban areas were also observed. These changes force delayed changes in classification and treatment protocols, implying a lack of health care system responsiveness [36]. Epidemiological studies of such special situations provide information on approaches to health care provision, enabling efficient allocation of health care services. Our study conducted an epidemiological investigation in a special setting (i.e., an orthopedic specialty hospital) in hopes of helping hospitals operate under similar environments allocate resources efficiently in the future.

To summarize, this retrospective cohort study was conducted on all patients who underwent orthopedic surgery at one hospital for approximately 10 years, and this study is significant because it targeted the largest number of patients over the longest period of time compared with previous studies. In particular, the most remarkable aspect in this study is that previous studies only used records from specialized hospitals for the reasons for transfer; hence, they were often recorded as symptoms or signs rather than diseases, such as chest pain, anemia, and hypotension. However, the present study is more meaningful because it identified as many specific diseases as possible in cooperation with the tertiary hospital to which the patients were transferred. Patients who were transferred to a tertiary hospital were recorded and followed up according to our hospital policy to prevent data omission. Furthermore, the possibility of errors in the records was minimized by separately storing the medical records of our doctors and the records transmitted from the tertiary hospital for research use. Nevertheless, because this was a single-institution study, the generalization of results may be limited. The lack of a control group, single-center design, and absence of long-term follow-up may limit the usefulness of this study. Potential misclassification error, problems in obtaining complete data from tertiary units, and lack of autopsies have been identified as limitations of this study. Given that this study only addressed transfers from orthopedic specialty hospital, our findings cannot be generalized to other hospitals with different infrastructures (ICUs, multi-professional settings, different health care systems, etc.).

## 5. Conclusions

The rate of transfer from the orthopedic hospital where this study was conducted to a tertiary hospital was very low, at 0.24%. Pulmonary embolism, acute cholecystitis, and cerebral infarct were the most common reasons for transfer, indicating that orthopedic hospitals must establish a system that can quickly respond to these causes. In particular, acute cholecystitis was the second most common reason for transfer, indicating the need for caution because elderly patients may experience only atypical clinical symptoms without abdominal pain or tenderness even the day after surgery.

## Figures and Tables

**Table 1:** Number of patients who underwent orthopedic surgery and procedures performed on patients transferred to tertiary hospitals.

Patients who underwent orthopedic surgery		67,118	
	TKA	28,556	42.5%
	THA	42	0.06%
Procedure performed on the transferred patient	TKA	136	81.4%
	THA	11	6.5%
	Shoulder	9	5.3%
	Ankle and foot	6	3.5%
	Etc.	5	2.9%

**Table 2:** Demographic characteristics and comorbidities of patients who underwent surgery.

Age		72.2 ± 9.2 (28–91)
Sex	Male	34 (20.3%)
	Female	133 (79.6%)
Height (cm)	Male	165.6 ± 4.7
	Female	151.5 ± 7.0
Weight (kg)	Male	67.5 ± 10.4
	Female	57.8 ± 11.1
BMI (kg/m^2^)	Male	24.5 ± 3.5
	Female	25.1 ± 4.2
Comorbidities	Hypertension	115
	Diabetes mellitus	36
	Hyperlipidemia	10
	Chronic obstructive pulmonary disease	9
	Hypothyroidism	9
	Heart failure	2
	Arrhythmia	1
	Obesity	75
Elixhauser Comorbidity Index		1.64 ± 1.14

**Table 3:** Number of patients transferred to tertiary hospitals by year from 2012 to 2022.

Year	Patients (*n*)	%
2012	11	6.6%
2013	10	6.0%
2014	8	4.8%
2015	15	9.0%
2016	30	18.0%
2017	18	10.8%
2018	27	16.2%
2019	25	15.0%
2020	14	8.4%
2021	4	2.4%
2022	5	3.0%
Total	167	100.0%

**Table 4:** Specific causes of transfer to a tertiary hospital.

Specific Causes	N.	%
Pulmonary embolism	27	16.2%
Acute cholecystitis	19	11.4%
Cerebral infarction	17	10.1%
Respiratory distress	9	5.3%
Myocardial infarction	8	4.7%
Intestinal obstruction or hemorrhage	7	4.1%
Surgical wound infection	6	3.5%
Pneumonia	5	2.9%
Change in the level of consciousness	4	2.3%
Chest pain	4	2.3%
Mental change	4	2.3%
Ventricular arrhythmia	3	1.7%
Heart failure	3	1.7%

**Table 5:** Classification by systematic category of reasons for transfer to tertiary hospitals.

Systematic Category	N.	%	Specific Causes
Venous thromboembolism	34	20.4%	Pulmonary embolism 27
			Fat embolism 5
			Deep vein thromboembolism 2
Heart	24	14.3%	Myocardial infarct 8
			Chest pain 4
			Heart failure 3
			Ventricular arrhythmia 3
			Angina, arrest, pacemaker problem, etc.
Brain	29	17.4%	Cerebral infarction 17
			Mental change 4
			Transient ischemic attack 2
Gastrointestinal	35	20.9%	Acute cholecystitis 19
			Intestinal hemorrhage 4
			Intestinal obstruction 3
			Acute appendicitis 3
			Peritonitis 2
			Abdominal pain, constipation, gastric ulcer, etc.
Lung	21	12.5%	Respiratory distress 9
			Pneumonia 5
			Pulmonary edema 3
			Asthma, etc.
Wound problem	10	6.0%	Surgical wound infection 6
			Refracture 2
			Dislocation 1
			Vessel injury 1
Sepsis	4	2.4%	
Etc.	10	5.9%	Abnormal LFT (liver function test) 3
			Abnormal renal function 2
			Unstable vital sign 2
			Hyponatremia 1
			Urethral obstruction 1
			Abdominal aorta aneurysm 1

**Table 6:** Number of patients transferred to tertiary hospitals and number of deaths by day of the week.

Day of the Week	N.	%	Death
Monday	21	12.5%	1
Tuesday	30	17.9%	1
Wednesday	36	21.5%	1
Thursday	26	15.5%	2
Friday	20	11.9%	3
Saturday	21	12.5%	0
Sunday	13	7.7%	3

## Data Availability

The data presented in this study are available upon reasonable request from the corresponding author.

## Abbreviations

The following abbreviations are used in this manuscript:

ASA	American Society of Anesthesiologists
TKA	Total Knee Arthroplasties
THA	Total Hip Arthroplasties
BMI	Body Mass Index
